# ﻿Phylogenetic studies on the genus *Candolleomyces* (Psathyrellaceae, Basidiomycota) occurring in the bed of the Indus River, Punjab, Pakistan, reveal three new species

**DOI:** 10.3897/mycokeys.112.141766

**Published:** 2025-01-16

**Authors:** Qirui Li, Muhammad Haqnawaz, Abdul Rehman Niazi, Abdul Nasir Khalid

**Affiliations:** 1 The High Efficacy Application of Natural Medicinal Resources Engineering Centre of Guizhou Province (The Key Laboratory of Optimal Utilization of Natural Medicine Resources), School of Pharmaceutical Sciences, Guizhou Medical University, Guiyang, Guizhou, 550004, China Guizhou Medical University Guizhou China; 2 Fungal Biology and Systematics Research Laboratory, Institute of Botany, University of the Punjab, Quaid-e-Azam Campus 54590, Lahore, Pakistan University of the Punjab Lahore Pakistan

**Keywords:** Caespitose, DNA barcoding, macrofungi, mushrooms, *Psathyrella*, systema­tics, terrestrial

## Abstract

During macrofungal surveys in 2019–2024, several specimens belonging to the family Psathyrellaceae were collected from the bed of the Indus River, Punjab, Pakistan. Phylogenetic analyses, based on ITS, LSU, and tef-1α sequences and morpho-anatomical study, confirmed the novelty and placement of three taxa in the genus *Candolleomyces*. They are described as *Candolleomycescrenatus*, *C.undulatus*, and *C.virgatus*. For distinguishing characters, *C.crenatus* has crenate cap margins, small basidiospores, and a marginate base of stipe. *Candolleomycesundulatus* has parabolic to campanulate, wavy margins, light purplish gray with a light brownish gray center of pileus, and an appendiculate, pendant annulus. *Candolleomycesvirgatus* has a parabolic to plane, distinct umbo, a virgate surface of pileus, 1–7 tiers, forking lamellae, and longitudinal striation on the surface of the stipe. Currently, *Candolleomyces* comprises 60 formally recognized species worldwide. However, with the inclusion of these three species, the total number rises to 63. Detailed descriptions, a phylogenetic estimate, morphological comparisons, and illustrations are provided.

## ﻿Introduction

Wächter and Melzer (2020) established the genus *Candolleomyces* D. Wächt. & A. Melzer (family Psathyrellaceae Locq.) to accommodate 25 species of *Psathyrella* (Fr.) Quél. It was typified through *Candolleomycescandolleanus* (Fr.) D. Wächt. & A. Melzer. The genus is characterized by small to large basidiomata, a veil that is mostly present as fibrillose, fugacious, scaly, or granulose; absence of pleurocystidia; and presence of clamp connections (Wächter and Melzer 2020). The members of the genus are mostly reported from terrestrial, lignicolous, and rarely fimicolous habitats. Genus *Candolleomyces* is separated from *Psathyrella* by phylogenetic analyses and by lacking pleurocystidia (Ahmad et al. 1997; Wächter and Melzer 2020; Asif et al. 2022; Haqnawaz et al. 2023b; Izhar et al. 2023; Ahmad et al. 2024; Haqnawaz et al. 2024a, b). Currently, the genus comprises 60 species and shows worldwide distribution (Ahmad et al. 1997; Wächter and Melzer 2020; Asif et al. 2022; Haqnawaz et al. 2023b; Izhar et al. 2023; Nayana and Pradeep 2023a, b; Ahmad et al 2024; Han et al. 2024; Haqnawaz et al. 2024a, b). Nevertheless, Asia is the richest continent for diversity of *Candolleomyces*, sporting forty-one species (68.33%), while eleven are known from Europe (18.33%), six from North America (10%), and one species each from Africa (1.66%) and South America (1.66%) (Haqnawaz et al. 2024a, b). From Pakistan, 14 species have been reported so far (Haqnawaz et al. 2024a, b), which corresponds to about a 34.14% contribution of the species known from Asia and 23.33% worldwide. In this study, based on the morpho-anatomical characters and phylogenetic analyses, we introduce three more new species of the genus *Candolleomyces*.

## ﻿Materials and methods

### ﻿Sampling site (Fig. 1)

Basidiomata of macrofungi were collected from different localities of the Indus Riverbed (30°25'21"N, 70°52'46"E) (129–139 m a.s.l.), Kot Addu, Punjab, Pakistan, during July to December 2019 to 2024. The highest temperature was 51 °C in summer, and the lowest temperature was -1 °C in winter, and the average annual rainfall is 127 mm (Ijaz et al. 2020; Haqnawaz et al. 2023a, b; Haqnawaz et al. 2024a, b). The predominant flora consists of *Vachellianilotica* (L.) P.J.H.Hurter & Mabb., *Saccharumbengalense* Retz., *S.spontaneum* L., *Dalbergiasissoo* Roxb., *Phoenixdactylifera* L., *Tamarixaphylla* (L.) Warb., *Albiziachinensis* (Osbeck) Merrill, and *Calotropisprocera* (Ait.) Ait., Hort. (Stewart 1972; Haqnawaz et al. 2024a, b).

**Figure 1.**
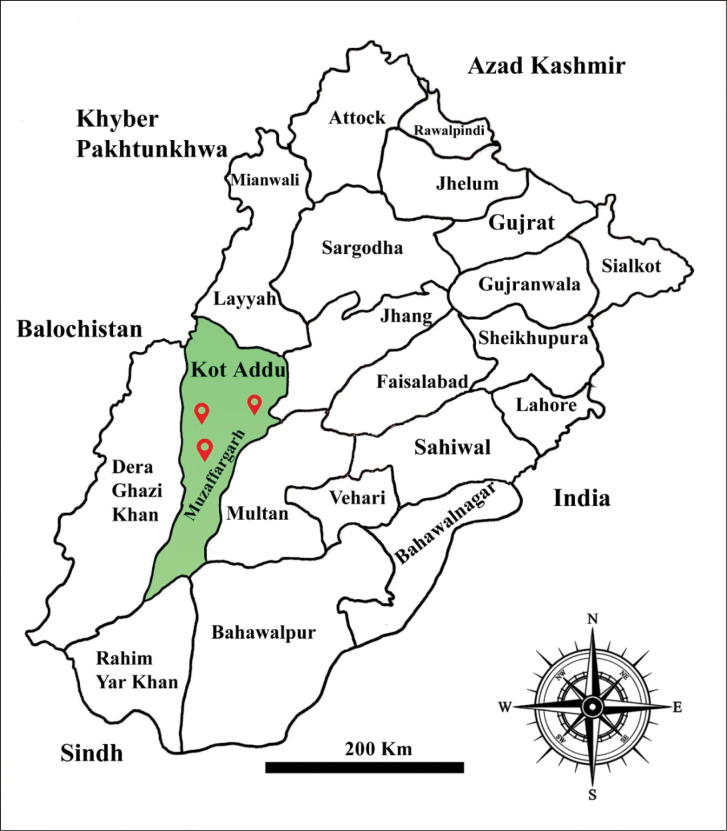
Map of sampling sites where the novel taxa were found, with the green color showing the district of the sampling sites.

### ﻿Morphological characterization

Fresh basidiomata were collected and photographed with tags. Morphological characteristics of samples were noted following terminologies by Vellinga (2001). Color codes were noted according to the Munsell Color Chart (1994). The collected basidiomata were dried using a hot air drier at 30 °C, packed in polythene bags, and deposited at the LAH Herbarium, Institute of Botany, University of the Punjab, Quaid-e-Azam Campus, Lahore, Pakistan. Tissues from different parts of the basidiomata were rehydrated in 5% KOH and stained in Congo Red (1% w/v). Microscopic structures were examined using a compound microscope (OLYMPUS BX43, Tokyo, Japan) and measured using ScopeImage 9.0 software connected to the microscope through a digital camera (HDCE–90D). The short form [n/m/p] indicates ‘n’ basidiospores measured from ‘m’ basidiomata of ‘p’ collections. Basidiospore measurements were recorded as (a–) b–c (–d), where a = extreme minimum value, range b–c covers at least 90% of the calculated values, and d = extreme maximum value; ‘Q’ shows the individual spore length/width ratio while ‘Qav’ presents the average of all Q values (Ge et al. 2010).

### ﻿Molecular phylogenetic analyses

Total DNA was extracted from lamellae of dried basidiomata using a modified 2% CTAB method (Bruns 1995), and the DNA quality was assessed by 1% (w/v) agarose gel electrophoresis (Voytas 2000). The ITS region of the rDNA was amplified using the ITS1F and ITS4 primers; for the LSU region, the LR0R and LR5 primers; and for the tef-1α gene, the EF1-983F and EF1-1567R primers were used (Vilgalys and Hester 1990; White et al. 1990; Gardes and Bruns 1993). The PCR procedure given by Usman and Khalid (2020) was followed, and PCR products were then sent to the sequencing company TsingKe, China.

The final ITS, LSU, and tef-1α rRNA consensus sequences were obtained by assembling both forward and reverse primers using BioEdit V 7.2.5 (Hall 2009). Sequences used in the combined dataset (ITS-LSU- and tef-1α rRNA) were retrieved from the NCBI database GenBank (Sayers et al. 2024), based on similarity of 95% identity or greater, plus all published sequences from the genus *Candolleomyces* (Ediriweera et al. 2023; Izhar et al. 2023; Nayana and Pradeep 2023a, b; Haqnawaz et al. 2024a, b). For the phylogenetic analyses, a Clustal W MUSCLE alignment (Edgar 2004) was implemented in BioEdit V 7.2.5 with manual adjustment. A combined (ITS-LSU-tef-1α) maximum likelihood phylogenetic tree was constructed using RAxMLHPC2 v. 8.2.12 on XSEDE tool (8.2.10) on the CIPRES portal online. The GTR+GAMMA nucleotide substitution model was used, and 1000 bootstrap iterations were performed with rapid bootstrapping (Miller 2010). Bayesian inference phylogenetic analyses were performed using MrBayes v. 3.2.1 (Ronquist et al. 2012, online version). The model of evolution was estimated by MrModeltest 2.2 (Nylander 2004). The Markov Chain Monte Carlo (MCMC) sampling in MrBayes v.3.2.2 (Ronquist et al. 2012) was used to determine the posterior probabilities (PP). Six simultaneous Markov chains were run for 1,000,000 generations, and trees were sampled every 1000^th^ generation. Bootstrap values ≥ 50% and Bayesian posterior probabilities (PP) ≥ 0.80 are mentioned on the branches that were visualized in FigTree v. 1.4.2 (Rambaut 2014). The newly generated sequences were deposited in GenBank, and short descriptions of the species were deposited in MycoBank (Robert et al. 2013).

## ﻿Results

### ﻿Phylogenetic analyses (Fig. 2, Table 1)

**Figure 2.**
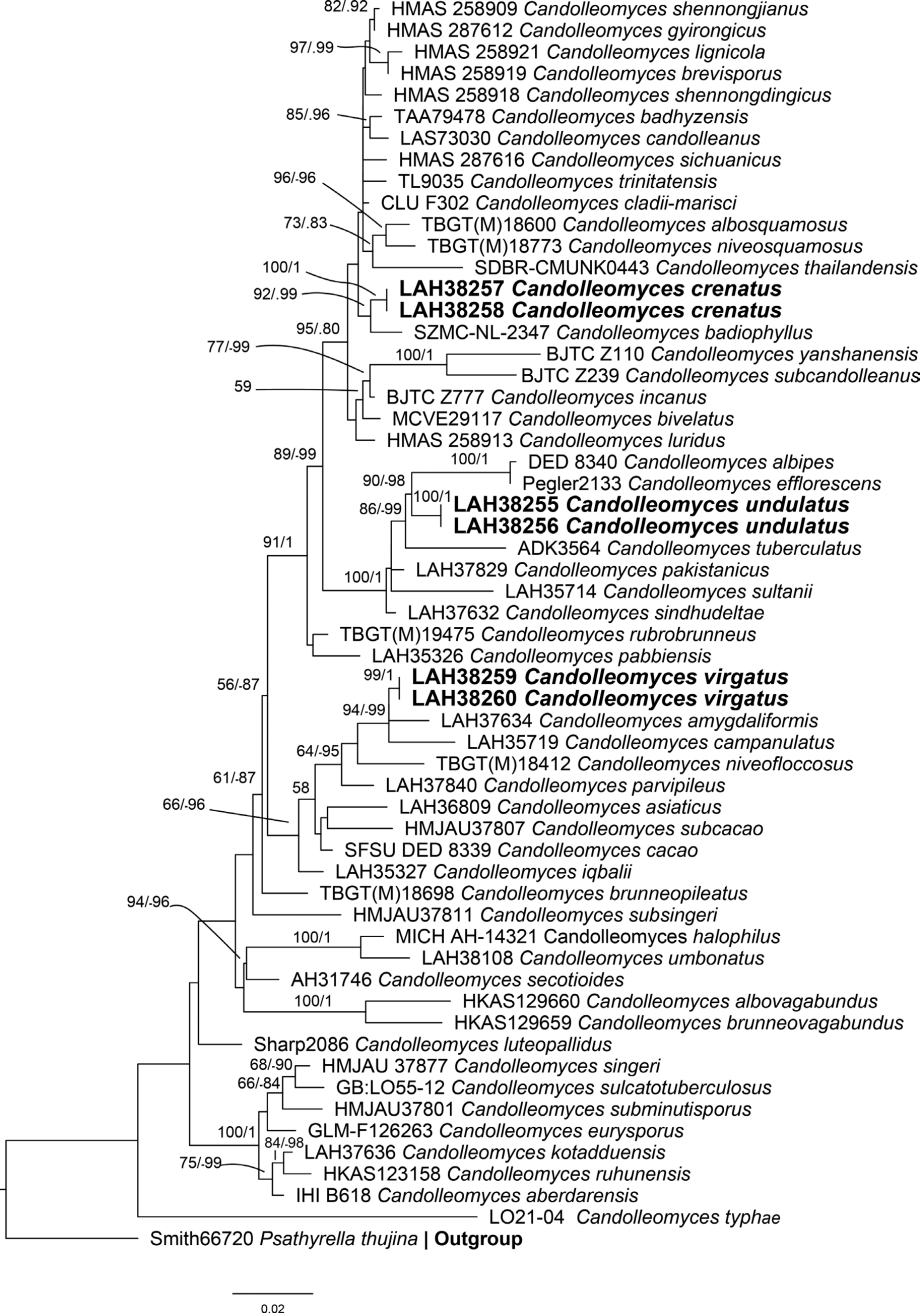
Phylogenetic tree of the genus *Candolleomyces* as generated by maximum likelihood (ML) and Bayesian analyses, based on combined ITS, LSU, and tef-1α sequences. Bootstrap values > 50%, based on 1,000 replicates and Bayesian posterior probabilities (PP) ≥ 0.80 are shown at the branches. Novel sequences, generated during this study, are shown in bold.

The combined phylogenetic tree was constructed based on ITS, LSU, and tef-1α regions using maximum likelihood and Bayesian inference methods. It contains 57 sequences of genus *Candolleomyces*, while the sequence of *Psathyrellathujina* A.H. Sm. was used as an outgroup. The newly proposed taxa are represented in bold in the final phylogenetic tree (Fig. 2). All three new species, viz., *Candolleomycescrenatus*, *C.undulatus*, and *C.virgatus*, formed separate branches from their closest species (Fig. 2). Our first taxon, *Candolleomycescrenatus*, makes a separate branch from *C.badiophyllus* with a difference of 17 base pairs in ITS, LSU, and tef-1α sequences. The second new species, *C.undulatus*, appears as a sister species to *C.efflorescens* (Sacc.) D. Wächt. & A. Melzer, *C.albipes* (Murrill) D. Wächt. & A. Melzer, and *C.tuberculatus* (Pat.) D. Wächt. & A. Melzer, with differences of 30, 33, and 35 base pairs in the ITS sequence, respectively. The third new species, *Candolleomycesvirgatus*, is a sister species to *C.campanulatus* A. Izhar, Z. Khan & Khalid and *C.amygdaliformis* Haqnawaz, Niazi, I. Ahmad & Khalid, with differences of 31 and 13 base pairs in the ITS-LSU sequence, respectively.

**Table 1.** Sequences used for phylogenetic analyses of *Candolleomyces*, including their current names, locality, voucher specimen, and GenBank accession numbers of the ITS, LSU, and tef-1α regions. Sequences generated during this study are shown in bold.

Species Name	Locality	Voucher	ITS	LSU	tef-1α
*Candolleomycesaberdarensis*	Kenya	IHI B618	MK421517	MK421517	-
*C.amygdaliformis* T	Pakistan	LAH37634	PP375293	PP375296	-
*C.albipes*	São Tomé	DED 8340	KX017209	-	-
*C.albosquamosus* T	India	TBGT(M)18600	OQ676549	-	-
*C.albovagabundus* T	China	HKAS129660	OR711041	OR711057	OR727285
*C.asiaticus* T	Pakistan	LAH36809	OK392605	OQ802842	-
*C.badhyzensis* T	Turkmenistan	TAA79478	KC992883	-	-
*C.badiophyllus*	Hungry	SZMC-NL-2347	FN430699	FM876268	FM897252
*C.bivelatus*	Italy	MCVE29117	MF325962	-	MF521811
*C.brunneopileatus* T	India	TBGT(M)18698	OQ878348	OR244401	-
*C.brunneovagabundus* T	China	HKAS129659	OR711031	OR711047	OR791600
*C.brevisporus* T	China	HMAS 258919	OR822167	OR822149	OR819986
*C.cacao* T	Sao Tomé	SFSU DED 8339	KX017210	-	-
*C.campanulatus* T	Pakistan	LAH35719	OQ308881	OQ802837	-
*C.candolleanus*	Sweden	LAS73030	KM030175	KM030175	-
*C.cladii-marisci* T	Italy	CLU F302	MK080112		-
***C.crenatus* T**	**Pakistan**	**LAH38257**	**PQ329548**	**PQ329553**	**PQ369432**
***C.crenatus***	**Pakistan**	**LAH38258**	**PQ329547**	**PQ329554**	**PQ369433**
*C.gyirongicus* T	China	HMAS 287612	PP734613	PP734624	PP729326
*C.kotadduensis* T	Pakistan	LAH37636	OQ968359	OQ968362	-
*C.lignicola* T	China	HMAS 258921	OR822169	OR822151	OR819988
*C.pakistanicus* T	Pakistan	LAH37829	OQ968356	OQ968363	-
*C.efflorescens*	Sri Lanka	Pegler2133	KC992941	-	-
*C.eurysporus* T	Vietnam	GLM-F126263	MT651560	MT651560	-
*C.halophilus* T	Spain	MICH AH-14321	MG825900	-	-
*C.incanus* T	China	BJTC Z777	ON042759	ON042766	ON098509
*C.iqbalii* T	Pakistan	LAH35327	OQ968353	OQ968366	-
*C.luteopallidus* T	USA	Sharp20863	KC992884	KC992884	-
*C.luridus* T	China	HMAS 258913	OR822160	OR822143	OR819980
*C.niveofloccosus* T	India	TBGT(M)18412	OQ878345	OR244387	-
*C.niveosquamosus* T	India	TBGT(M)18773	PP741631	PP741635	-
*C.pabbiensis* T	Pakistan	LAH35326	PP058339	PP058345	-
*C.parvipileus* T	Pakistan	LAH37840	OQ968357	OR506278	-
*C.rubrobrunneus* T	India	TBGT(M)19475	PP741633	PP741636	-
*C.ruhunensis* T	Sri Lanka	HKAS123158	ON685315	-	-
*C.secotioides* T	Mexico	AH31746	KR003281	KR003282	KR003283
*C.shennongjianus* T	China	HMAS 258909	OR822157	OR822139	OR819976
*C.shennongdingicus* T	China	HMAS 258918	OR822166	OR822148	OR819985
*C.sindhudeltae* T	Pakistan	LAH37632	OQ247908	OQ247912	-
*C.singeri*	China	HMJAU 37877	MW301073	MW301091	MW314080
*C.subcacao* T	China	HMJAU37807	MW301064	MW301092	MW314081
*C.sichuanicus* T	China	HMAS 287616	PP734617	PP734628	PP729330
*C.subcandolleanus* T	China	BJTC Z239	ON042755	ON042762	ON098505
*C.subminutisporus* T	China	HMJAU37801	MW301066	MW301094	MW314083
*C.subsingeri* T	China	HMJAU37811	MG734715	MW301097	MW314085
*C.sulcatotuberculosus*	Germany	GB:LO55-12	KJ138422	-	-
*C.sultanii* T	Pakistan	LAH35714	OQ308835	OQ801565	-
*C.thailandensis* T	Thailand	SDBR-CMUNK0443	MZ146874	-	-
*C.trinitatensis*	Ecuador	TL9035	KC992882	-	-
*C.tuberculatus*	Benin	ADK3564	KC992934	-	-
*C.typhae*	Sweden	LO21-04	DQ389721	DQ389721	KJ732776
*C.* umbonatus T	Pakistan	LAH38108	PP058341	PP058347	-
***C.undulatus* T**	**Pakistan**	**LAH38255**	**PQ329546**	**PQ329551**	**PQ369428**
***C.undulatus***	**Pakistan**	**LAH38256**	**PQ329545**	**PQ329552**	**PQ369429**
***C.virgatus* T**	**Pakistan**	**LAH38259**	**PQ329543**	**PQ329549**	**PQ369430**
***C.virgatus***	**Pakistan**	**LAH38260**	**PQ329544**	**PQ329550**	**PQ369431**
*C.yanshanensis* T	China	BJTC Z110	ON042758	ON042765	ON098507
*Psathyrellathujina* **(Outgroup)**	USA	Smith66720	KC992876	KC992876	-

### ﻿Taxonomy

#### ﻿ Candolleomyces crenatus

Taxon classificationFungiAgaricalesPsathyrellaceae

Haqnawaz, Niazi & Khalid sp. nov.

9D2265BE-B962-5278-AEBD-6D408EE554CE

 855774

Figs 3 , 4

##### Etymology.

Species name “*crenatus*” (Latin) refers to the crenate cap margins.

##### Holotype.

Pakistan • Punjab Province: Kot Addu District, Indus Riverbed, (30°28'03"N, 70°49'25"E, 126 m a.s.l.), on loamy soil, rich in organic matter, under *Vachellianilotica*, 07 Aug. 2022, Muhammad Haqnawaz, Q–13 (***Holotype*** LAH38257). GenBank: PQ329548 [ITS], PQ329553 [LSU], PQ369432 [tef-1α].

**Figure 3.**
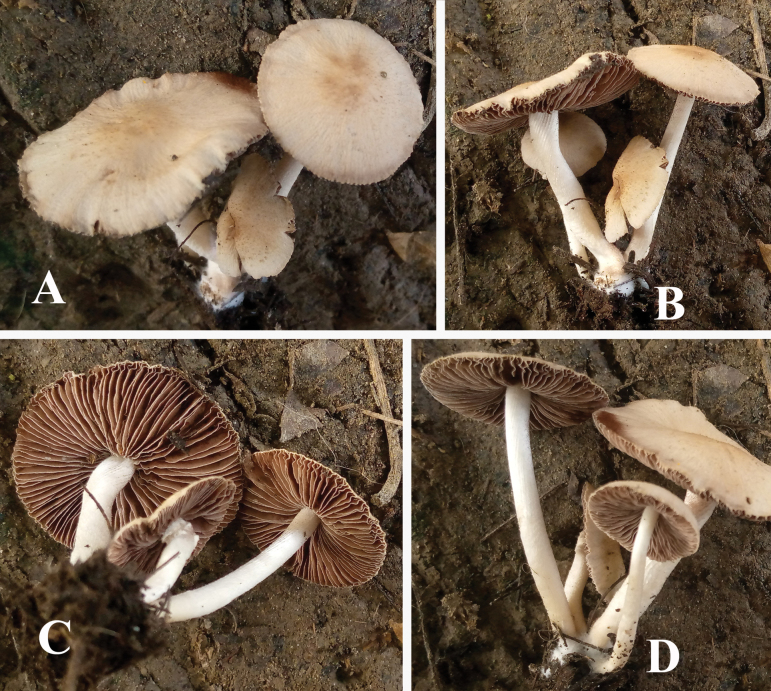
**A–D** basidiomata of *Candolleomycescrenatus* sp. nov. (Holotype LAH38257). Scale bars: 10 mm.

##### Diagnosis.

This species is different from *Candolleomycesbadiophyllus* by its convex to plane, crenate cap margins, wrinkled, dull surface, light gray, with dull orange center of pileus, marginate base of stipe, and small basidiospores (7–9 × 4–5 µm).

**Figure 4.**
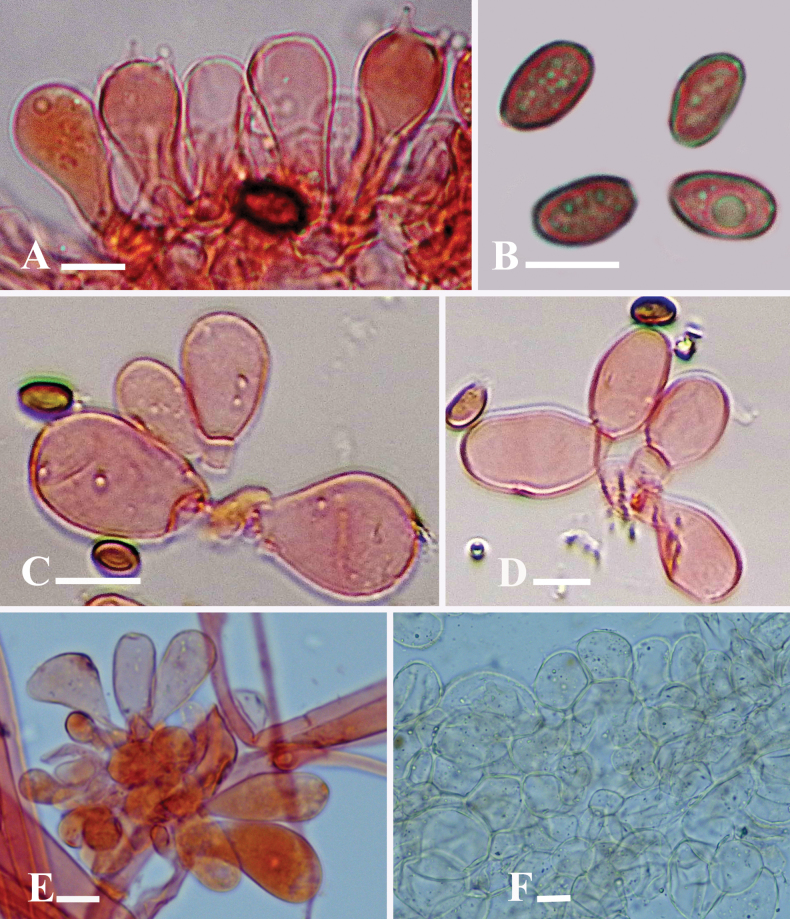
Microscopic structures of *Candolleomycescrenatus* sp. nov. (Holotype LAH38257) **A** basidia **B** basidiospores **C, D** cheilocystidia **E** caulocystidia **F** pileipellis. Scale bars: 10 µm (**A–F**).

##### Description.

***Pileus*** 14–34 mm diameter, convex to plane, straight shape, and crenate cap margins; presence of veil elements; wrinkled, dull surface; light gray (7.5 YR 8/1) with dull orange (7.5YR 7/3) center. ***Lamellae*** adnexed, ventricose, average, close, fimbriate, unequal, 1–5 tiers, dull reddish brown (5 YR 5/3). ***Stipe*** 23–35 × 3–5 mm, compressed, flexuous, tomentose, marginate base. ***Annulus*** absent. Taste and odor are not recorded.

***Basidiospores*** [150/3/2], (7–)7.5–8.5(–9) × (4–)4.5–5(–5.7) µm, Q = 1.4–1.8, Qav = 1.61, ellipsoid, thick-walled, smooth, rarely guttulate, central germpore present, reddish brown in water. ***Basidia*** (15–)16–23(–24) × (7–)7.5–11(–12) µm, clavate to broadly clavate, hyaline to yellowish in water, thick-walled, smooth, with 3–4 sterigmata. ***Cheilocystidia*** (15–)16–31(–32) × (7–)8–12(–13) µm, ellipsoid to broadly ellipsoid, clavate to broadly clavate, globose, utriform to utriform with median constriction, cylindrical, hyaline to yellowish in water, thick-walled. ***Pleurocystidia*** absent. ***Pileipellis*** an irregular epithelium, thick-walled, 18–30 × 16–24 µm, globose to subglobose and ellipsoid to broadly ellipsoid, clavate to broadly clavate cells, hyaline to light yellowish in water. ***Stipitipellis*** a cutis, hyphae subregular, branched 6–17 µm in diameter, thin-walled, septate, and hyaline to yellow in water. ***Caulocystidia*** (17–)18–42(–43) × (8–)9–14(–15) µm, utriform, clavate to broadly clavate, globose, broadly ellipsoid, hyaline in water. Clamp connections present in all tissues.

##### Ecology and habitat.

Terrestrial, caespitose, on loamy soil rich in organic matter, under *Vachellianilotica*.

##### Additional material examined.

Pakistan • Punjab Province: Kot Addu, Pirhar Gherbi, (30°25'07"N, 70°52'35"E, 136 m a.s.l.), on loamy soil, 11 Aug. 2023, Muhammad Haqnawaz, HA–03 (***Paratype*** LAH38258). GenBank: PQ329547 [ITS], PQ329554 [LSU], PQ369433 [tef-1α].

#### ﻿ Candolleomyces undulatus

Taxon classificationFungiAgaricalesPsathyrellaceae

Haqnawaz, Niazi & Khalid sp. nov.

B767CB0C-FDDE-5509-9FF6-F7DC8D43C4B9

 855775

Figs 5 , 6

##### Etymology.

Species name “*undulatus*” (Latin) refers to the wavy cap margins.

##### Holotype.

Pakistan • Punjab Province: Kot Addu District, Indus Riverbed, (30°25'33"N, 70°52'12"E, 130 m a.s.l.), on loamy soil, rich in organic matter, under *Phoenixdactylifera*, 28 Aug. 2020, Muhammad Haqnawaz, KA–39 (***Holotype*** LAH38255). GenBank: PQ329546 [ITS], PQ329551 [LSU], PQ369428 [tef-1α].

**Figure 5.**
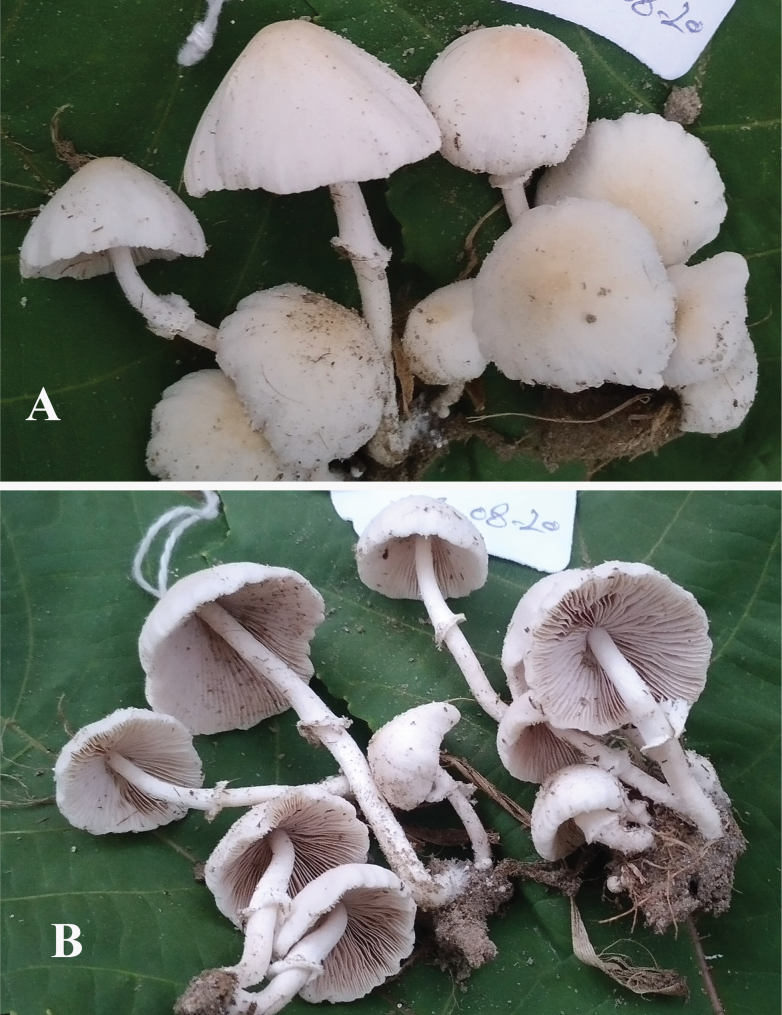
**A, B** basidiomata of *Candolleomycesundulatus* sp. nov. (Holotype LAH38255). Scale bars: 10 mm.

##### Diagnosis.

This species is different from its closest species by parabolic to campanulate, wavy margins, light purplish gray with light brownish gray center of pileus, appendiculate, pendant annulus, oblong to ellipsoid, globose, ovoid, cheilocystidia, and caulocystidia.

**Figure 6.**
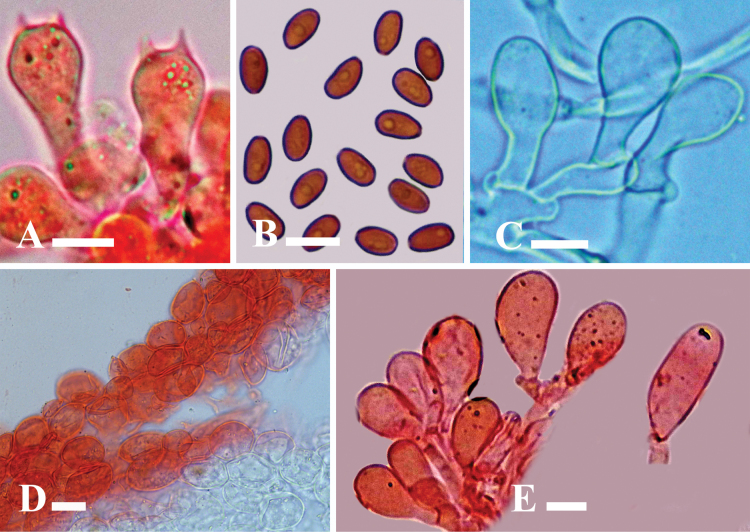
Microscopic structures of *Candolleomycesundulatus* sp. nov. (Holotype. LAH38255) **A** basidia **B** basidiospores **C** caulocystidia **D** pileipellis **E** cheilocystidia. Scale bars: 10 µm (**A–E**).

##### Description.

***Pileus*** 10–27 mm diameter, parabolic to campanulate, straight shape and wavy cap of margins, presence of veil elements, light purplish gray (5 P 7/1), and light brownish gray (5YR 7/2) center. ***Lamellae*** adnexed to adnate, narrow, average, close, eroded, unequal, 1–3 tiers, light reddish gray (7.5 R 7/1), ***Stipe*** 15–46 × 2–4 mm, concolourous to pileus, punctate, flexuous, tomentose. ***Annulus*** appendiculate and pendant. ***Taste*** and ***odor*** not recorded.

***Basidiospores*** [150/3/2], (6–)6.5–7.5(–8) × (3.5–)4–5(–6.6) µm, Q = 1.15–1.94, Qav = 1.58, ellipsoid to oblong, thick-walled, smooth, guttulate, germpore present, brownish with yellowish guttule in water. ***Basidia*** (15–)16–24(–25) × (7–)7.5–11(–12) µm, clavate to broadly clavate, rarely cylindrical, spherical with broad peduncle, hyaline in water, thick-walled, smooth, with 2–4 sterigmata. ***Cheilocystidia*** (15–)16–31(–32) × (7–)8–12(–13) µm, oblong to ellipsoid, globose, ovoid, utriform, hyaline in water, thick-walled. ***Pleurocystidia*** absent. ***Pileipellis*** an irregular epithelium, thick-walled, 18–30 × 16–24 µm diameter, globose to subglobose and ellipsoid to broadly ellipsoid, hyaline in water. ***Stipitipellis*** a cutis, hyphae subregular, branched 7–18 µm in diameter, thin-walled, septate, hyaline in water. ***Caulocystidia*** 17–)18–42(–43) × (8–)9–14(–15) µm, utriform, clavate to broadly clavate, ovoid, spheropedunculate, globose, hyaline in water. Clamp connection present in all tissues.

##### Ecology and habitat.

Terrestrial, connate, on loamy soil rich in organic matter, under *Phoenixdactylifera*.

##### Additional material examined.

Pakistan • Punjab Province: Kot Addu, Noor Shah Thal, (30°27'39"N, 70°52'40"E, 129 m a.s.l.), on loamy soil, 11 Aug 2021, Muhammad Haqnawaz, SSC–44 (***Paratype*** LAH38256). GenBank: PQ329545 [ITS], PQ329552 [LSU], PQ369429 [tef-1α].

#### ﻿ Candolleomyces virgatus

Taxon classificationFungiAgaricalesPsathyrellaceae

Haqnawaz, Niazi & Khalid sp. nov.

25AC64DF-00CF-5404-A087-DD1248A654FD

 855776

Figs 7 , 8

##### Etymology.

Species name “*virgatus*” (Latin) refers to the virgate pileus surface.

##### Holotype.

Pakistan • Punjab Province: Kot Addu District, Indus Riverbed, (30°14'44"N, 70°51'04"E, 134 m a.s.l.), on soil, rich in organic matter, 29 Aug 2020, Muhammad Haqnawaz, EF–17 (***Holotype*** LAH38259). GenBank: PQ329543 [ITS], PQ329549 [LSU], PQ369430 [tef-1α].

**Figure 7.**
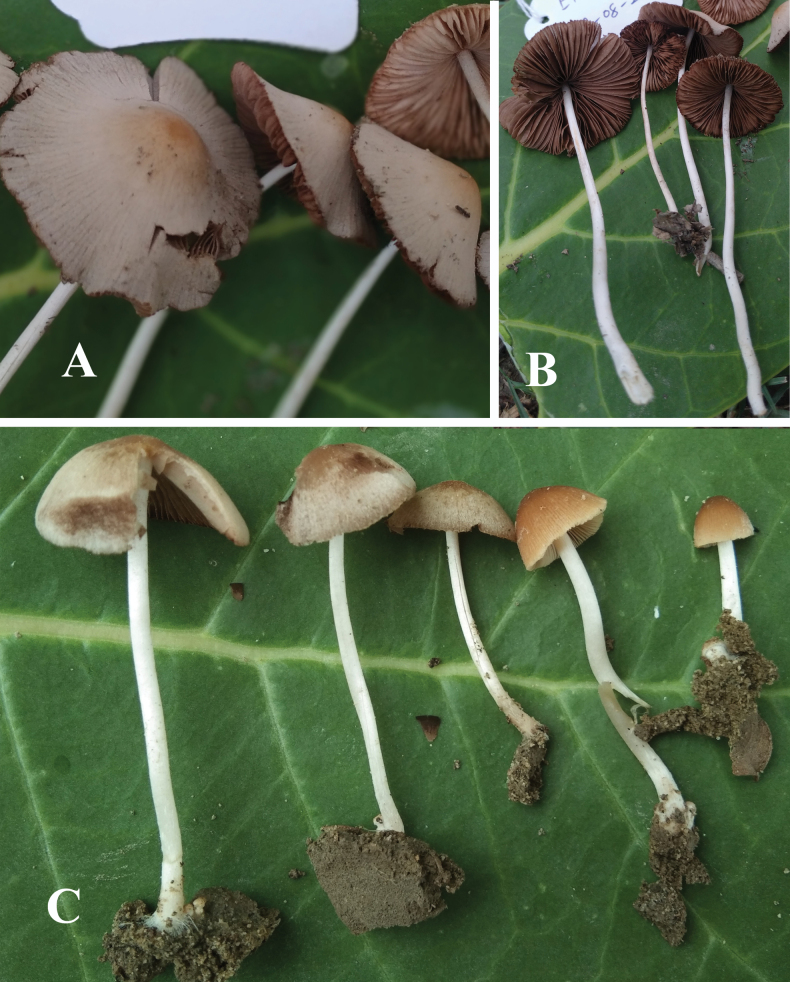
**A–C** basidiomata of *Candolleomycesvirgatus* sp. nov. (Holotype LAH38259). Scale bars: 10 mm.

##### Diagnosis.

This species is different from *Candolleomycescampanulatus* by its parabolic to plane, distinct umbo, virgate surface of pileus, crinkled, 1–7 tiers of forking lamellae, marginate base, longitudinally striation on surface of stipe, lageniform with moniliform at top, moniliform, cylindrical, tibiiform, conical cheilocystidia.

**Figure 8.**
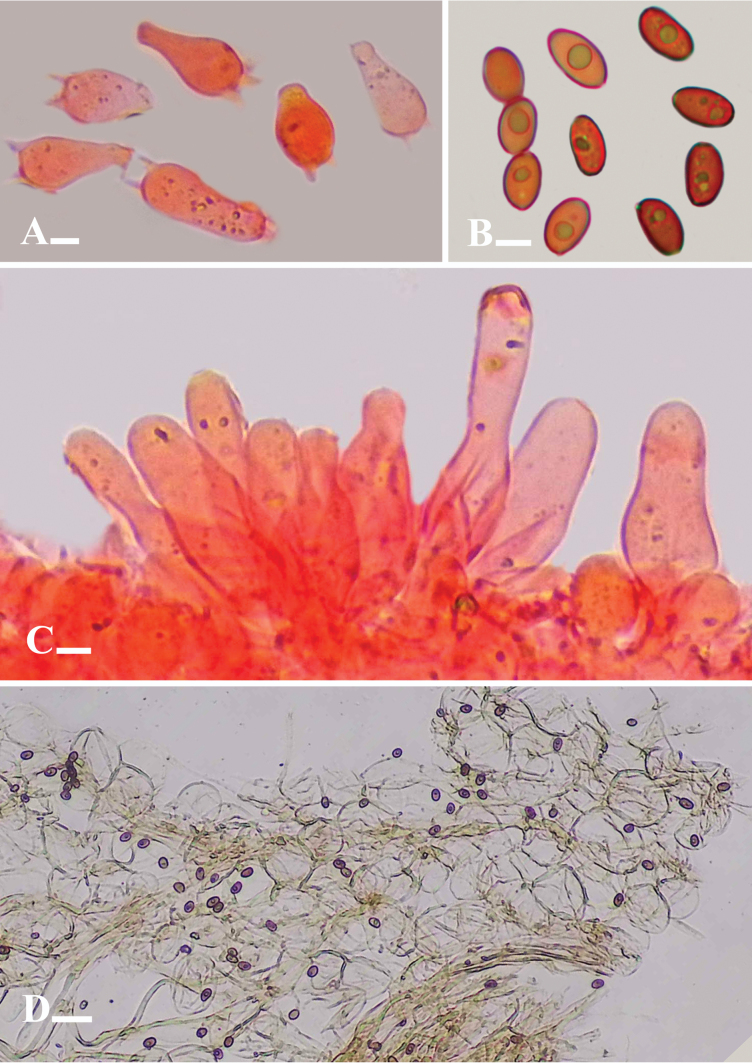
**A–D** microscopic structures of *Candolleomycesvirgatus* (Holotype. LAH38259) **A** basidia **B** basidiospores **C** cheilocystidia **D** pileipellis. Scale bars: 10 µm (**A–C**); (**D**) 20 µm (**D**).

##### Description.

Basidiomata medium-sized. ***Pileus*** 17–30 mm diameter, parabolic at young stage, campanulate to plane, umbonatus, straight shape and regular cap of margins, virgate, light brownish gray (7.5 YR 7/1), and dull reddish brown (5YR 5/4) center. ***Lamellae*** adnate, broad, crinkled, 1–7 tiers of lamellae, branched or forking; some are not branched, grayish red (10R 6/2). ***Stipe*** 40–80 × 1–4 mm, white gray (N 8/0), slightly flexuous, hollow, tomentose, and broad at base, marginate, longitudinally striation. Annulus absent.

***Basidiospores*** [150/2/3], (6–)7–9(–10) × (4–)4.5–5(–6) µm, Q = 1.2–1.8, Qav = 1.61, ellipsoid to broadly ellipsoid, thick-walled, smooth, guttulate, germ pore present, purplish to brownish with greenish to yellow guttule in 5% KOH and water. ***Basidia*** (12–)13–29(–30) × (6–)7–9(–10) µm, clavate, broadly clavate, hyaline to light yellowish in 5% KOH, thick-walled, smooth, with 2–4 sterigmata. ***Cheilocystidia*** (26–)27–46(–47) × (8–)9–13(–14) µm, utriform, lageniform with moniliform at top, moniliform, cylindrical, tibiiform, conical, hyaline to light yellowish in 5% KOH. ***Pleurocystidia*** and ***Caulocystidia*** absent. ***Pileipellis*** an irregular epithelium; pileipellis cells 30–60 × 20–42 µm diameter, globose to subglobose and ellipsoid to broadly ellipsoid, clavate, hyaline to light yellowish in 5% KOH. ***Stipitipellis*** a cutis, hyphae subregular, branched 6–15 µm in diameter, thin-walled, septate, hyaline to light yellowish in 5% KOH. Clamp connection present in all tissues.

##### Ecology and habitat.

Terrestrial, gregarious, scattered, on loamy soil rich in organic matter.

##### Additional material examined.

Pakistan • Punjab Province: Kot Addu, Noor Shah Thal, (30°27'47"N, 71°02'05"E, 132 m a.s.l.), on loamy soil, 02 September 2020, Muhammad Haqnawaz, KA–59 (***Paratype***; LAH38260) GenBank PQ329544 [ITS], PQ329550 [LSU], PQ369431 [tef-1α].

## ﻿Discussion

In this study, three new species of *Candolleomyces* are being introduced using morpho-anatomical and phylogenetic analyses. It is very interesting that all new species, *Candolleomycescrenatus*, *C.undulatus*, and *C.virgatus*, formed separate branches from their closest species. Morphologically, *Candolleomycescrenatus* is distinguished from its closest species, *C.badiophyllus*, due to its convex to plane, crenate cap margins, wrinkled, dull surface, light gray, with a dull orange center of pileus, a marginate base of stipe, and small basidiospores (7–9 × 4–5 µm). *Candolleomycesbadiophyllus*, a French species, differs from new taxon due to its subglandiform to bell shaped, ochreous yellow, silken, shiny surface of pileus, bulbous base of stipe, and larger basidiospores (11–14.5 × 5–6.2 µm) (Romagnesi 1952).

*Candolleomycesundulatus* differs from its sister species, *C.efflorescens*, due to its parabolic to campanulate pileus, light reddish gray lamellae, brown basidiospores, terrestrial, connate, and its occurren under *Phoenixdactylifera*. While *C.efflorescens*, from Sri Lanka, differs due to its hemispleuric pileus, white lamellae, and brown-purple basidiospores. It occurred on dead mossy trunks under *Peradenyaceylon* (Saccardo 1887). The second closest species, *C.albipes*, from Jamaica, differs due to its convex to broadly convex, incurved margins of pileus, clavate cheilocystidia, and absence of caulocystidia (Desjardin and Perry 2016). Similarly, *C.tuberculatus*, a French taxon, differs from our newly described species due to its globose, greenish, and loaded with scales in the shape of small rounded tubercles on the pileus, membranous annulus, and purple, ovoid basidiospores (Patouillard 1885–1903).

According to the phylogenetic analyses (Fig. 2), *Candolleomycesvirgatus* forms a separate branch from *C.amygdaliformis* and *C.campanulatus*. Morpho-anatomically, *C.virgatus* is different due to its parabolic to plane shape, distinct umbo, virgate surface of pileus, crinkled, 1–7 tiers of forking lamellae, marginate base, longitudinal striations on the surface of the stipe, and lageniform with moniliform at the top, moniliform, cylindrical, tibiiform, and conical cheilocystidia. While *C.amygdaliformis* is different from *C.virgatus* by its conical pileus with absence of umbo, 1–3 tiers of lamellae, shiny surface of stipe, amygdaliform to phaseoliform basidiospores, and ovoid-pedunculate cheilocystidia (Ahmad et al. 2024). *Candolleomycescampanulatus* differs from *C.virgatus* by its conical to hemispheric, indistinct umbo, fibrillose, and plain dry surface of pileus, eroded, 1–2 tiers of lamellae, crystals, or mucoid droplets at the apices of cheilocystidia (Izhar et al. 2023).

These findings show that the diversity of the genus *Candolleomyces* in Pakistan is much more than expected. Hence, many more systematic surveys are required to explore and understand the diversity and distribution the genus.

## Supplementary Material

XML Treatment for Candolleomyces crenatus

XML Treatment for Candolleomyces undulatus

XML Treatment for Candolleomyces virgatus

## ﻿Additional information

### Author ORCIDs

Muhammad Haqnawaz https://orcid.org/0000-0003-4667-242X

Qirui Li https://orcid.org/0000-0001-8735-2890

Abdul Rehman Niazi https://orcid.org/0000-0002-1118-1148

Abdul Nasir Khalid https://orcid.org/0000-0002-5635-8031

### Data availability

All of the data that support the findings of this study are available in the main text or Supplementary Information.

## Supplementary materials

Supplementary material 1 Fasta file of phylogenetic data Data type fas https://binary.pensoft.net/file/1221201This dataset is made available under the Open Database License (http://opendatacommons.org/licenses/odbl/1.0/). The Open Database License (ODbL) is a license agreement intended to allow users to freely share, modify, and use this Dataset while maintaining this same freedom for others, provided that the original source and author(s) are credited.Author: Muhammad Haqnawaz

## References

[B1] Ahmad S Iqbal S Khalid AN (1997) Fungi of Pakistan. Sultan Ahmad Mycological Society of Pakistan, Department of Botany, University of the Punjab, Quaid-e-Azam campus, Lahore 248 pp.

[B2] Ahmad I Haqnawaz M Niazi AR Khalid AN (2024) A new species of *Candolleomyces* (Psathyrellaceae) from Pakistan. Phytotaxa 658(3): 280–288. 10.11646/phytotaxa.658.3.6

[B3] Asif M Izhar A Niazi AR Khalid AN (2022) *Candolleomycesasiaticus* sp. nov. (Psathyrellaceae, Agaricales), is a novel species from Punjab, Pakistan. European Journal of Taxonomy 826: 176–187. 10.5852/ejt.2022.826.1845

[B4] Bhunjun CS Niskanen T Suwannarach N Wannathes N Chen YJ McKenzie EH Maharachchikumbura SSN Buyck B Zhao C-L Fan Y-G Zhang J-Y Dissanayake AJ Marasinghe DS Jayawardena RS Kumla J Padamsee M Chen Y-Y Liimatainen K Ammirati JF Phukhamsakda C Liu J-K Phonrob W Randrianjohany É Hongsanan S Cheewangkoon R Bundhun D Khuna S Yu W-J Deng L-S Lu Y-Z Hyde KD Lumyong S (2022) The numbers of fungi: are the most speciose genera truly diverse? Fungal Diversity 114(1): 387–462. 10.1007/s13225-022-00501-4

[B5] Bruns TD (1995) Thoughts on the processes that maintain local species diversity of ectomycorrhizal fungi. Plant Soil 170: 63–73. 10.1007/BF02183055

[B6] Desjardin DE Perry BA (2016) Dark-spored species of Agaricineae from Republic of São Tomé and Príncipe, West Africa. Mycosphere 7(3): 359–391. 10.5943/mycosphere/7/3/8

[B7] Edgar RC (2004) MUSCLE: multiple sequence alignment with high accuracy and high throughput. Nucleic acids research 32(5): 1792–1797. 10.1093/nar/gkh340 15034147 PMC390337

[B8] Ediriweera AN Voto P Karunarathna SC Kumla J Thiyagaraja V Wijesooriya MK Jianchu X (2023) A new species and a new record in the Agaricales from Sri Lanka. Mycological Observations 6: 94.

[B9] Gardes M Bruns TD (1993) ITS primers with enhanced specificity for basidiomycetes-application to the identification of mycorrhizae and rusts. Molecular Ecology 2(2): 113–118. 10.1111/j.1365-294X.1993.tb00005.x 8180733

[B10] Ge ZW Yang ZL Vellinga EC (2010) The genus *Macrolepiota* (Agaricaceae, Basidiomycota) in China. Fungal Diversity 45(1): 81–98. 10.1007/s13225-010-0062-0

[B11] Hall TA (1999) BioEdit: a user-friendly biological sequence alignment editor and analysis program for windows 95/98/ NT/7. Nucleic Acids Symposium Series 41: 95–98.

[B12] Haqnawaz M Niazi AR Khalid AN (2023a) Two new species of *Xanthagaricus* from Punjab, Pakistan. Phytotaxa 583(2): 163–173. 10.11646/phytotaxa.583.2.4

[B13] Haqnawaz M Niazi AR Khalid AN (2023b) A study on the genus *Candolleomyces* (Agaricales: Psathyrellaceae) from Punjab, Pakistan. BMC Microbiology 23(1): 1–8. 10.1186/s12866-023-02938-2 37434121 PMC10334618

[B14] Haqnawaz M Usman M Javaid A Ramzan F Bibi A Niazi AR Naseer A Afshan NS Khalid AN (2024a) Four new species of *Candolleomyces* (Psathyrellaceae) from the Punjab Plains, Pakistan. Mycologia 1–13. 10.1080/00275514.2024.2374208 39137793

[B15] Haqnawaz M Usman M Fatima N Niazi AR Khalid AN (2024b) Three new species of the genus *Candolleomyces* (Psathyrellaceae) from Pakistan. Plant Systematics and Evolution 310(6): 42. 10.1007/s00606-024-01925-y

[B16] Han XX Phurbu D Ma GF Li YZ Mei YJ Liu DM Lin F-C Zhao R-L Thongklang N Cao B (2024) A Taxonomic Study of *Candolleomyces* Specimens from China Revealed Seven New Species. Journal of Fungi 10(7): 499. 10.3390/jof10070499 39057384 PMC11277608

[B17] Ijaz M Ahmad HR Bibi S Ayub MA Khalid S (2020) Soil salinity detection and monitoring using Landsat data: a case study from Kot Addu, Pakistan. Arabian Journal of Geosciences 13(13): 1–9. 10.1007/s12517-020-05572-8

[B18] Izhar A Asif M Khan Z Khalid AN (2023) Introducing two new members of the genus *Candolleomyces* (Agaricales, Psathyrellaceae) from Punjab, Pakistan. Plant Systematics and Evolution 309(5): 40. 10.1007/s00606-023-01876-w

[B19] Miller MA Pfeiffer W Schwartz T (2010) Creating the CIPRES Science Gateway for inference of large phylogenetic trees. In: gateway Computing Environments Workshop (gCE). New Orleans, LA, 8 pp. 10.1109/GCE.2010.5676129

[B20] Munsell A (1994) Soil color charts. Revised edition. New York: Macbeth Division of Kollmorgen Instruments Corporation.

[B21] Nayana PK Pradeep CK (2023a) A new species of *Candolleomyces* (Psathyrellaceae, Agaricales) from Western Ghats, India. Phytotaxa 606(1): 63–72. 10.11646/phytotaxa.606.1.6

[B22] Nayana PK Pradeep CK (2023b) New species and new record of *Candolleomyces* (Psathyrellaceae) from India. Botany 101(11): 472–484. 10.1139/cjb-2023-0066

[B23] Nayana PK Pradeep CK (2024) Morphological and phylogenetic analyses reveal two new species of *Candolleomyces* (Psathyrellaceae) from India. Phytotaxa 659(3): 255–267. 10.11646/phytotaxa.659.3.3

[B24] Nylander JAA (2004) MrModeltest v2. Program distributed by the author. Evolutionary Biology Centre, Uppsala University, Uppsala.

[B25] Patouillard N (1885–1903) Champignons de la Guadelou: in Bulletin de la Société mycologique de France by Société mycologique de France, 196 pp.

[B26] Rambaut A (2014) FigTree 1.4. 2 software. Institute of Evolutionary Biology, University of Edinburgh. Rambaut, A., Suchard, M.A. & Xie, D. Tracer v. 1.6. http://beast.bio.ed.ac.uk/Tracer [Accessed 9 January 2018]

[B27] Robert V Vu D Amor ABH van de Wiele N Brouwer C Jabas B Szoke S Dridi A Triki M ben Daoud S Chouchen O Vaas L de Cock A Stalpers JA Stalpers D Verkley GJM Groenewald M Borges dos Santos F Stegehuis G Li W Wu L Zhang R Ma J Zhou M Gorjón SP Eurwilaichitr L Ingsriswang S Hansen K Schoch C Robbertse B Irinyi L Meyer W Cardinali G Hawksworth DL Taylor JW Crous PW (2013) MycoBank gearing up for new horizons. IMA fungus 4: 371–379. 10.5598/imafungus.2013.04.02.16 24563843 PMC3905949

[B28] Romagnesi H (1952) Species et formae ex genere Drosophila Quélet. Publications de la Société Linnéenne de Lyon 21(6): 151–156. 10.3406/linly.1952.7506

[B29] Ronquist F Teslenko M Van Der Mark P Ayres DL Darling A Höhna S Larget B Liu L Suchard MA Huelsenbeck JP (2012) MrBayes 3.2: Efficient Bayesian phylogenetic inference and model choice across a large model space. Systematic Biology 61: 539–542. 10.1093/sysbio/sys029 22357727 PMC3329765

[B30] Saccardo PAS (1887) Fungorum, Vol .5. Agaricineae. P. A, Saccardo. Padua, 1067 pp.

[B31] Sayers EW Beck J Bolton EE Brister JR Chan J Comeau DC Connor R DiCuccio M Farrell CM Feldgarden M Fine AM Funk K Hatcher E Hoeppner M Kane M Kannan S Katz KS Kelly C Klimke W Kim S Kimchi A Landrum M Lathrop S Lu Z Malheiro A Marchler-Bauer A Murphy TD Phan L Prasad AB Pujar S Sawyer A Schmieder E Schneider VA Schoch CL Sharma S Thibaud-Nissen F Trawick BW Venkatapathi T Wang J Pruitt KD Sherry ST (2024) Database resources of the national center for biotechnology information. Nucleic Acids Research 52(D1): D33. 10.1093/nar/gkad1044 PMC10767890 37994677

[B32] Sicoli G Passalacqua NG De Giuseppe AB Palermo AM Pellegrino G (2019) A new species of *Psathyrella* (Psathyrellaceae, Agaricales) from Italy. MycoKeys 52: 89–102. 10.3897/mycokeys.52.31415 31148934 PMC6533211

[B33] Stewart RR (1972) An annotated catalogue of the vascular plants of West Pakistan and Kashmir. In: Nasir E, Ali SI (Eds) Flora of Pakistan. Fakhri Printing Press, Karachi.

[B34] Usman M Khalid AN (2020) *Termitomycesacriumbonatus* sp. nov. (Lyophyllaceae, Agaricales) from Pakistan. Phytotaxa 477(2): 217–228. 10.11646/phytotaxa.477.2.6

[B35] Vellinga EC (2001) Agaricaceae. In: Noordeloos ME, Kuyper TW, Vellinga EC (Eds) Flora Agaricina Neerlandica 5. Rotterdam: A.A. Balkema Publishers 220 pp.

[B36] Vilgalys R Hester M (1990) Rapid genetic identification and mapping of enzymatically amplified ribosomal DNA from several Cryptococcus species. Journal of Bacteriology 172: 4238–4246. 10.1128/jb.172.8.4238-4246.1990 2376561 PMC213247

[B37] Voytas D (2000) Agarose gel electrophoresis. Current protocols in molecular biology 51(1): 2–5. 10.1002/0471142727.mb0205as51 34266220

[B38] Wächter D Melzer A (2020) Proposal for a subdivision of the family Psathyrellaceae based on a taxon-rich phylogenetic analysis with iterative multigene guide tree. Mycological Progress 19(11): 1151–1265. 10.1007/s11557-020-01606-3

[B39] White TJ Bruns T Lee S Taylor J (1990) Amplification and direct sequencing of fungal ribosomal RNA genes for phylogenetics. In: PCR protocols: a guide to methods and applications. Academic Press, San Diego 482 pp. 10.1016/B978-0-12-372180-8.50042-1

